# Ferumoxytol-enhanced MR venography for diagnosis of venous thoracic outlet syndrome

**DOI:** 10.1016/j.radcr.2023.03.052

**Published:** 2023-04-29

**Authors:** Christopher Lan, Mohammad H. Madani, Anjali Pawar, Lorenzo Nardo, Ahmadreza Ghasemiesfe

**Affiliations:** a School of Medicine, University of California, Davis, 4610 X St, Sacramento, CA 95817, USA; b Department of Radiology, University of California, Davis, 4860 Y St, Suite 3100, Sacramento, CA 95817, USA; c Department of Pediatrics, University of California, Davis, 2521 Stockton Blvd, Sacramento, CA 95817, USA

**Keywords:** Ferumoxytol, Venous, Thoracic, Outlet, Contrast, Venography

## Abstract

Venous thoracic outlet syndrome commonly results in arm swelling and pain as the subclavian vein is obstructed within the thoracic inlet. We report the use of ferumoxytol-enhanced contrast MRI in the diagnosis of venous thoracic outlet syndrome in a male adolescent. In this patient who presented with right upper extremity thrombosis, ferumoxytol-enhanced MRI of the chest was able to show both chronic subclavian vein thromboses and dynamic occlusion of the subclavian veins with arm abduction consistent with Paget-Schroetter syndrome.

## Introduction

Thoracic outlet syndrome (TOS) results from the compression of the neurovasculature through the thoracic inlet. Venous thoracic outlet syndrome commonly results in arm swelling and pain due to subclavian vein obstruction and can present with or without thrombosis [1]. In this case report, an adolescent male with an upper right extremity deep vein thrombosis (DVT) is diagnosed with TOS by MRI despite having no known risk factors. Ferumoxytol has emerged as a newer alternative to gadolinium-based contrast agents for MRIs, as it is mainly confined to the intravascular compartment upon administration, has a long intravascular half-life of 14-15 hours, and provides a longer period for higher resolution, higher signal-to-noise vascular imaging [2]. While used in other vasculature studies such as to evaluate thoracic central vein stenosis [3], this is the first reported usage of ferumoxytol in a dynamic study to evaluate for TOS.

## Case report

A 15-year-old man presented to the ED with a red, swollen, and tender right arm associated with fever and headache for 3 days. The swelling was more pronounced in his right forearm and improved with straightening of his elbow. He was found to have a right-upper-extremity DVT by duplex ultrasound. The right upper extremity showed acute, occluding, intraluminal echoes in the axillary, brachial, and basilic veins to the elbow. Absent Doppler and color filling were also noted in the axillary, brachial, and basilic veins to the elbow level. He had no known history of injury or overuse to the extremity. Prothrombin and Factor V Leiden mutation testing were both negative. DRVTT ratio was slightly elevated at 1.26 (<1.2 is normal). There was no family history of clotting disorders, strokes, or heart attacks. He was anticoagulated and discharged with enoxaparin 2 days later. Because his DVT was unprovoked, there was suspicion for thoracic outlet syndrome. One month after discharge, he received an MRA of the chest to evaluate for thoracic outlet syndrome.

**Fig. 1:**
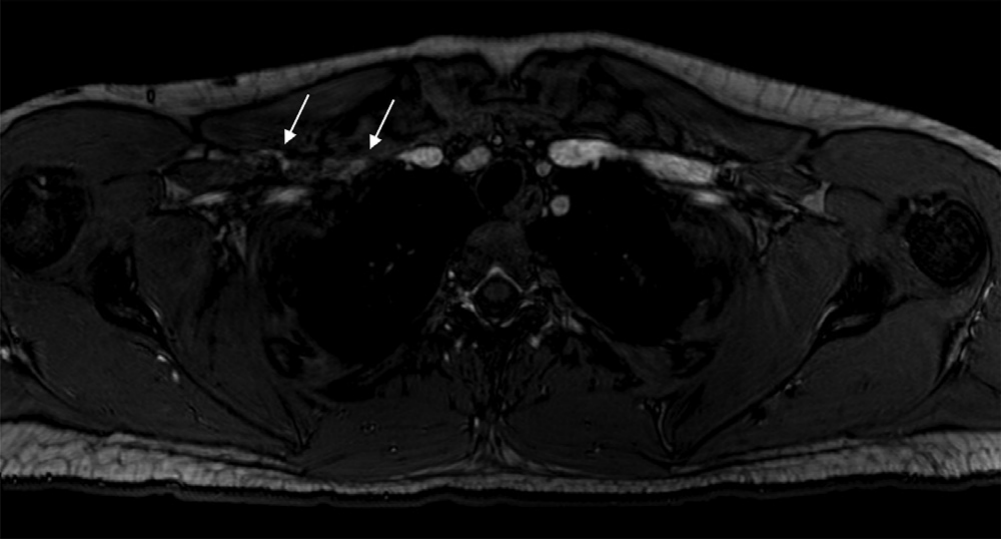
Filling defects in the right subclavian vein (white arrows) with arms in the downward position, consistent with the patient's duplex ultrasound findings of a right-sided deep vein thrombosis.

**Fig. 2:**
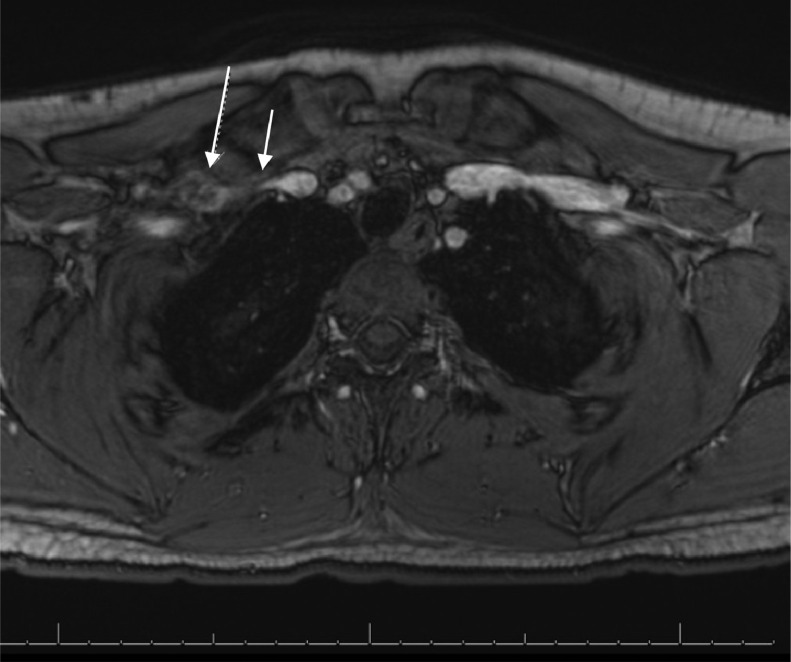
Occlusion of the right subclavian vein (small white arrow) in the abducted/upward arm position at the thoracic inlet. The large arrow demonstrates the partial thrombosis of the right subclavian vein.

**Fig. 3:**
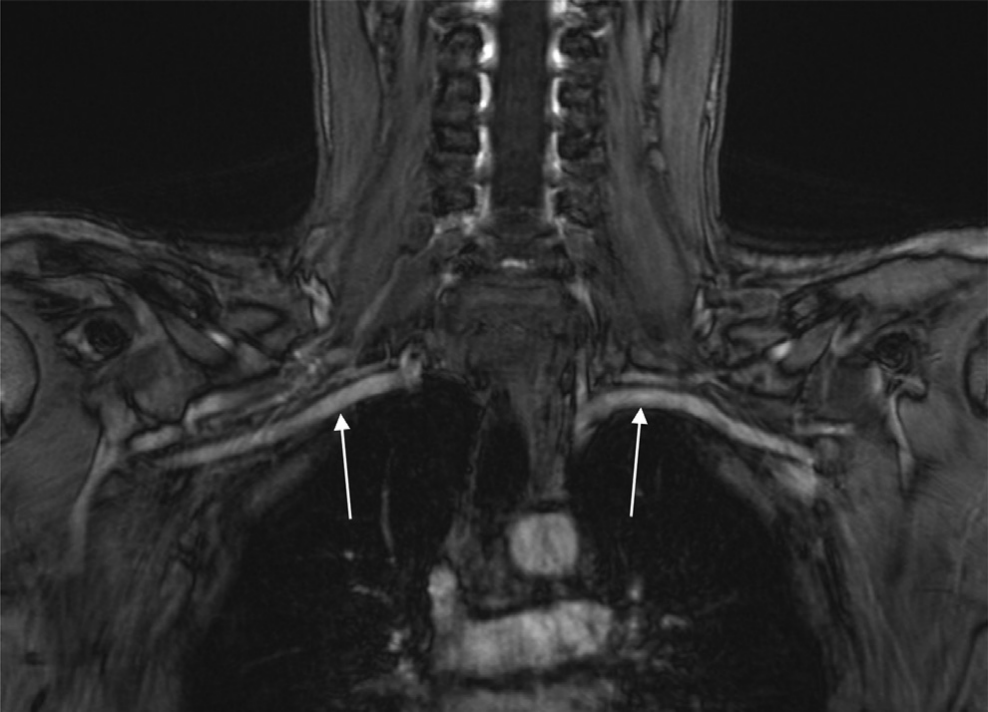
Subclavian arteries (white arrows) initially visualized in the adducted/downward positions of the arms. The right subclavian artery measures 5.9 mm and the left subclavian artery measures 5.7 mm; these are relatively symmetric and nonstenotic compared to the abducted positioning in Fig. 4.

**Fig. 4:**
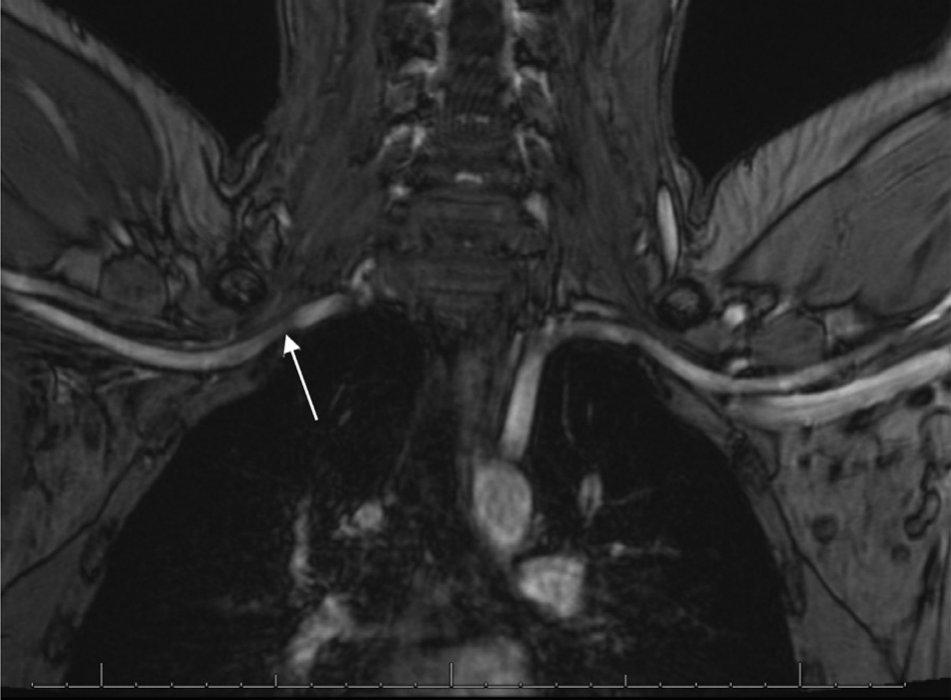
Dynamic narrowing of the right subclavian artery (white arrow) is demonstrated with arms in the abducted/upward positions. The diameter of the right subclavian artery narrows down to 4.2 mm while the left artery diameter remains constant at 5.7 mm.

A multiplanar, multisequence MRA of the chest was performed with both arms in abducted and adducted positions. Postcontrast images were obtained following the uneventful intravenous administration of ferumoxytol 3 mg/kg (7 mL). The MRA enhancement showed linear intraluminal filling defects within the right subclavian and axillary veins, compatible with chronic thrombosis (Figs. 1 and 2). The right subclavian vein was moderately narrowed at the level of thoracic inlet with his right arm in an adducted, relaxed position. There was complete dynamic occlusion of the right subclavian vein at the level of thoracic inlet between the clavicle and first rib in an abducted arm position. The left subclavian vein also demonstrated dynamic occlusion in the abducted position. The left subclavian vein was patent without filling defects in the relaxed, adducted left arm images. Both of his subclavian arteries were patent. However, there was mild narrowing of his proximal right subclavian artery at the crossing superior to the right first rib with a endoluminal diameter of 4 mm with abducted arms versus 6 mm on adducted arms (Figs. 3 and 4). In conclusion, the dynamic occlusion of the bilateral subclavian veins at the thoracic inlet in an abducted arm position was consistent with venous thoracic outlet syndrome.

Two weeks after the MRI, the patient was evaluated by pediatric hematology/oncology. He had been adherent to his Lovenox injections twice a day. His upper right extremity remained swollen compared to his left, and he was transitioned to oral anticoagulation with apixaban for another 6-12 weeks.

## Discussion

Diagnosis of TOS can often prove challenging. Furthermore, subclassification of TOS as venous TOS, arterial TOS, and neurogenic TOS lacks practice guidelines due to an absence of unified diagnostic workup, which often leads to treatment uncertainty [4]. Paget-Schroetter syndrome refers to effort-induced subclavian vein thrombosis associated with subclavian vein compression at the thoracic inlet, which presents more in younger males [5]. In this version of venous TOS, treatment options have a wide range and include anticoagulation, surgical decompression, thrombolysis and thrombectomy. Improved imaging and diagnostic reporting can potentially lead to a consistent treatment guideline for venous TOS.

Contrast-enhanced MRA with gadopentetate dimeglumine and gadobenate dimeglumine have been shown to allow excellent imaging of the venous structures of the arm in patients with suspected TOS, as well as evaluating for restenosis or residual vascular compression postsurgery. However, one limitation of gadolinium-based contrast-enhanced MRA includes the need for patient repositioning and 2 separate injections for arm abduction and arm rest positions [6]. Ferumoxytol serves as a blood pool MRA agent with a long intravascular half-life of greater than 14 hours. In our case, we were able to use a single ferumoxytol injection for dynamic arm testing of TOS through contrast-enhanced MRA (Fig. 1, Fig. 2, Fig. 3, Fig. 4).

## Patient consent

Written informed consent has been obtained from the legal guardian of the patient, who is a minor at the time of submission. We express appreciation for the cooperation of the patient and the patient's family.

Patient's legal representative has provided permission to publish the medical record pertinent to this case report which is documented. We may provide legal proof of this consent if requested by any authority or *Radiology Case Reports*.
